# Deficiency of Acute-Phase Serum Amyloid A Exacerbates Sepsis-Induced Mortality and Lung Injury in Mice

**DOI:** 10.3390/ijms242417501

**Published:** 2023-12-15

**Authors:** Ailing Ji, Andrea C. Trumbauer, Victoria P. Noffsinger, Luke W. Meredith, Brittany Dong, Qian Wang, Ling Guo, Xiangan Li, Frederick C. De Beer, Nancy R. Webb, Lisa R. Tannock, Marlene E. Starr, Christopher M. Waters, Preetha Shridas

**Affiliations:** 1 Saha Cardiovascular Research Center, University of Kentucky, Lexington, KY 40536, USA; ailing.ji@uky.edu (A.J.); acma242@g.uky.edu (A.C.T.); victoria.noffsinger@uky.edu (V.P.N.); luke.meredith@uky.edu (L.W.M.); qian.wang@uky.edu (Q.W.); ling.guo@uky.edu (L.G.); xli2@email.uky.edu (X.L.); nrwebb1@uky.edu (N.R.W.); lisa.tannock@uky.edu (L.R.T.); 2 Department of Physiology, University of Kentucky, Lexington, KY 40536, USA; brittany.dong@uky.edu (B.D.); chris.waters41@uky.edu (C.M.W.); 3 Pharmacology and Nutritional Sciences, University of Kentucky, Lexington, KY 40536, USA; marlene.starr@uky.edu; 4 Department of Internal Medicine, University of Kentucky, Lexington, KY 40536, USA; fcdebe1@uky.edu; 5 Department of Surgery, College of Medicine, University of Kentucky, Lexington, KY 40536, USA

**Keywords:** serum amyloid A, sepsis, acute-phase response, lung injury, neutrophil infiltration, inflammation

## Abstract

Serum amyloid A (SAA) is a family of proteins, the plasma levels of which may increase >1000-fold in acute inflammatory states. We investigated the role of SAA in sepsis using mice deficient in all three acute-phase SAA isoforms (SAA-TKO). SAA deficiency significantly increased mortality rates in the three experimental sepsis mouse models: cecal ligation and puncture (CLP), cecal slurry (CS) injection, and lipopolysaccharide (LPS) treatments. SAA-TKO mice had exacerbated lung pathology compared to wild-type (WT) mice after CLP. A bulk RNA sequencing performed on lung tissues excised 24 h after CLP indicated significant enrichment in the expression of genes associated with chemokine production, chemokine and cytokine-mediated signaling, neutrophil chemotaxis, and neutrophil migration in SAA-TKO compared to WT mice. Consistently, myeloperoxidase activity and neutrophil counts were significantly increased in the lungs of septic SAA-TKO mice compared to WT mice. The in vitro treatment of HL-60, neutrophil-like cells, with SAA or SAA bound to a high-density lipoprotein (SAA-HDL), significantly decreased cellular transmigration through laminin-coated membranes compared to untreated cells. Thus, SAA potentially prevents neutrophil transmigration into injured lungs, thus reducing exacerbated tissue injury and mortality. In conclusion, we demonstrate for the first time that endogenous SAA plays a protective role in sepsis, including ameliorating lung injury.

## 1. Introduction

Sepsis is a life-threatening condition, and the third International Consensus (Sepsis-3) currently defines sepsis as “organ dysfunction caused by a dysregulated host response to infection” [1]. So far, despite over 100 randomized controlled trials, no drugs have shown survival benefits in sepsis treatment [2]. This is partially a consequence of our limited understanding of the extremely complex mechanisms that regulate the inflammatory response. Identifying molecules involved in sepsis, especially endogenous protective modulators, is important in understanding the related mechanisms and providing new insights for efficient therapies.

The acute-phase response (APR) begins between 12 and 48 h after the initiation of inflammation. The APR is regulated by two major players, glucocorticoids and interleukin-6 (IL-6). Many acute-phase proteins are hardly detectable in healthy mammals but are induced 10–1000-fold during inflammation. However, their function in sepsis remains unclear [3]. Serum amyloid A (SAA) is a family of acute-phase proteins with serum concentrations rising dramatically during acute inflammation. The human genome encodes two acute-phase SAA proteins, SAA1 and SAA2. Mice encode a third conserved acute-phase *SAA* gene called *Saa3*. *Saa3* is a pseudogene in humans due to an early stop codon [4]. Human *SAA1* and *SAA2* are 96% homologous over their entire length and correspond to mouse *Saa1.1* and *Saa2.1*. The SAA3 protein is similar in general structure but with distinct sequence differences [5,6]. Here, we use “SAA” to denote the three mouse acute-phase SAA proteins, SAA1.1, SAA2.1, and SAA3. Acute-phase SAA is predominantly synthesized by the liver and is secreted into plasma, where it binds to high-density lipoprotein (HDL) [7]. The present work suggests that SAA is protective in sepsis, and SAA may be involved in the resolution of inflammation by regulating neutrophil infiltration into injured organs. 

## 2. Results

### 2.1. Whole-Body SAA Deficiency Aggravates Sepsis-Induced Mortality 

**Figure 1:**
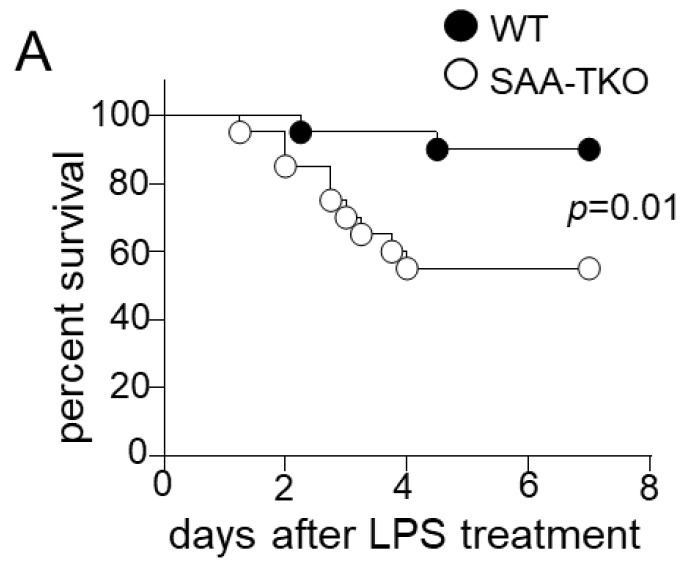

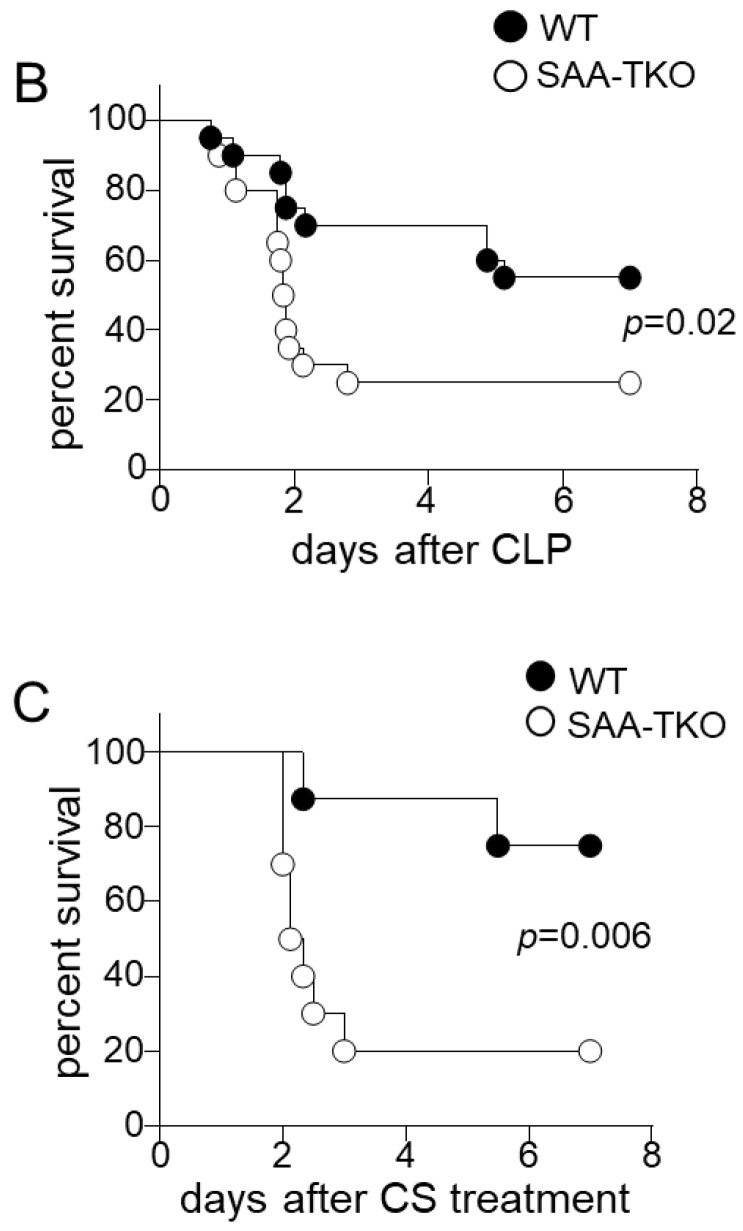
SAA protects mice against sepsis death. Survival was monitored for 7 days in WT and SAA-TKO mice following: (**A**) treatment with lethal LPS (7.5 mg/kg; male mice *n* = 20/group); (**B**) cecal-ligation and puncture CLP (male and female mice; *n* = 10/sex/group); and (**C**) cecal slurry (CS) treatment (female mice; *n* = 8–10 mice/group), for which mice received antibiotic treatment (imipenem IPM, 1.5 mg i.p.) and fluid resuscitation (s.c) beginning 12 h after the CS injection and continued twice daily as described under “Section 4”.

To evaluate the role of SAA in sepsis, we used two mouse strains, wild-type C57BL/6 (WT) and C57BL/6 mice, deficient in all three inducible isoforms of SAA (SAA-TKO)—SAA1.1, SAA2.1 and SAA3. Multiple mouse sepsis models were adopted to establish the results: (1) treatment with a lethal dose of lipopolysaccharide (LPS); (2) cecal ligation and puncture (CLP [8]); and (3) injection of cecal slurry (CS [9]). Intraperitoneal administration of LPS (i.p. 7.5 mg/kg) led to 10% mortality in WT mice and 42% in SAA-TKO mice (*p* = 0.01; Figure 1A). Interestingly, in mice subjected to CLP, mortality was only 45% in WT mice (9/20) but 75% (15/20) in SAA-TKO mice until 7 days (*p* = 0.02; Figure 1B). We did not observe any sex differences in the phenotypes of SAA-TKO mice. Consistent results were obtained in the CS model: there was a significant increase in mortality in SAA-TKO mice (80%) compared to WT mice (29%) (*p* = 0.006; Figure 1C). As expected, in all the three models studied, the SAA levels increased significantly following sepsis. In mice subjected to CLP, the levels of SAA mRNA in the livers of WT mice at 24 h after CLP were ~8-fold higher than the levels at 4 h (Figure S1A). As expected, no SAA expression was observed in the livers of SAA-TKO mice following CLP (Figure S1A). Following CS treatment, the plasma SAA levels peaked at 48 h (16.6 ± 3.9 mg/mL) and decreased gradually, reaching 0.95 mg/mL by the 7th day of sepsis (Figure S1B). 

### 2.2. SAA Is Dispensable for Endotoxin and Bacterial Clearance or Systemic Proinflammatory Responses

**Figure 2:**
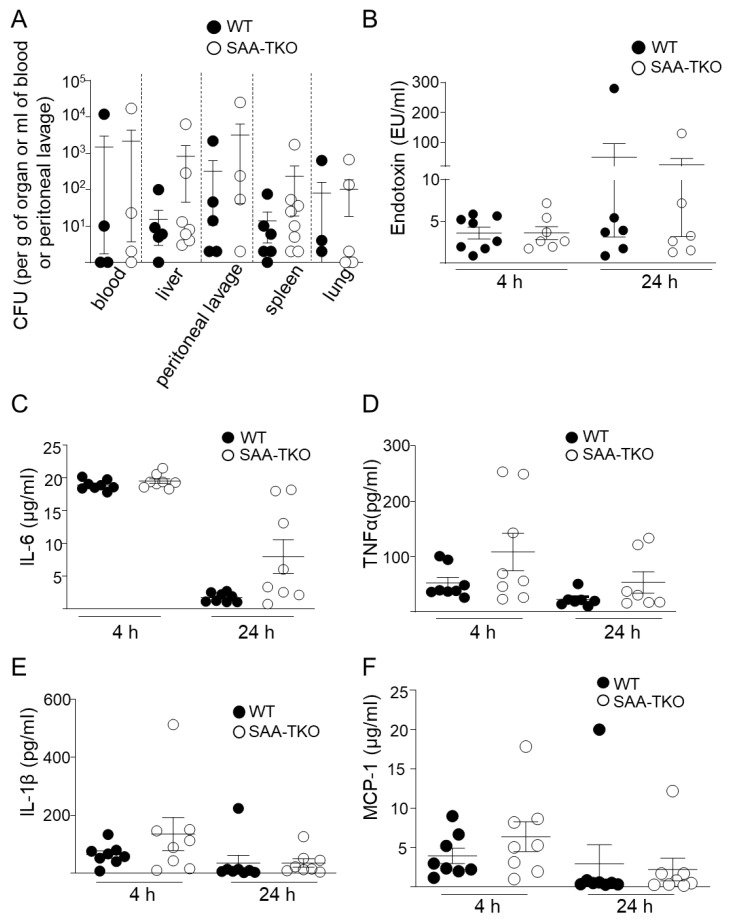
Deficiency of SAA does not significantly impact bacterial clearance and endotoxin or plasma cytokine levels in sepsis. (**A**) Number of bacterial colony forming units (CFU) in the indicated organs, blood, or peritoneal lavage in WT and SAA-TKO mice 24 h after CLP, determined as described under “Section 4”. (**B**) Plasma endotoxin levels in WT and SAA-TKO mice 4 h and 24 h after CLP, determined as described under “Section 4”. (**C**–**F**) Plasma IL-6, TNFα, IL-1β, and MCP-1 in WT and SAA-TKO mice at 4 h and 24 h after CLP, determined as described under “Section 4”. Data are mean ± SEM.

In a previous study using mice with inducible transgenic expressions of human SAA1, Cheng et al. reported that SAA1 acts as an LPS-binding protein and helps clear endotoxins in circulation, resulting in decreased systemic inflammatory responses [10]. However, in the CLP model, bacterial titers measured 24 h after CLP in the blood, peritoneal lavage, liver, spleen, and lungs in SAA-TKO mice were not different from those in WT mice (Figure 2A). Consistently, the plasma endotoxin levels were not significantly different between SAA-TKO and WT mice at 4 h or 24 h following CLP (Figure 2B). We measured proinflammatory cytokines and found no significant differences in serum levels between WT and SAA-TKO mice (shown in Figure 2C–F are IL-6, TNFα, IL-1β, and MCP-1, respectively), although there was a trend toward increased levels in SAA-TKO mice at 4 h for TNFα, IL-1β, and MCP-1 and at 24 h for IL-6 and TNFα after CLP (Figure 2C–F). Consistently, upon analyzing a panel of serum cytokine and chemokine levels, no significant differences were observed between WT and SAA-TKO mice at both 4 h or 24 h following CLP (Supplementary Table S1). Consistent with the observations in the CLP model, in mice treated with a sublethal dose of LPS (3 mg/mL), there was no significant difference in the plasma endotoxin levels (Figure S2A) between WT and SAA-TKO at 24 h after treatment. Plasma IL-6, TNFα, and IL-1β did not differ significantly at 2 h, 12 h, and 24 h after LPS treatment between WT and SAA-TKO mice (Figure S2B–D). Thus, it appears that endogenous SAA does not play a significant role in endotoxin clearance or systemic inflammatory responses in mice during sepsis.

### 2.3. Deficiency of SAA Does Not Change Plasma HDL-Cholesterol (HDL-C) Levels in Mice during Sepsis

**Figure 3:**
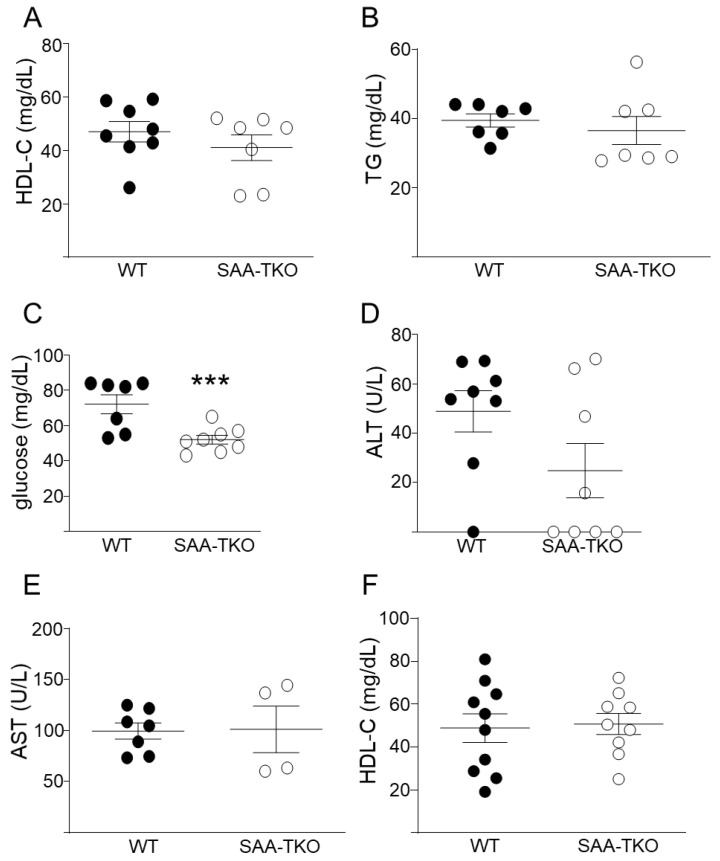
SAA deficiency does not alter plasma HDL-C levels. (**A**) plasma HDL-C (**B**) plasma triglycerides, (**C**) blood glucose, (**D**,**E**) plasma ALT and AST levels 24 h after CLP in WT and SAA-TKO mice, and (**F**) plasma HDL-C 24 h after LPS treatment as described under “Section 4”. Data are mean ± SEM, *** = *p* < 0.001.

SAA enrichment is believed to reduce HDL-cholesterol (HDL-C) concentrations during acute inflammation [11,12]. However, studies from our lab indicated that SAA does not impact the plasma HDL-C or apoA1 levels in mice [13,14]. Consistent with our earlier observation [13,14], there was no significant difference in the HDL-C levels between WT and SAA-TKO 24 h after CLP (Figure 3A). There was no significant difference in plasma triglycerides (TG) in SAA-TKO mice compared to WT mice (Figure 3B). The blood glucose levels were significantly lower in SAA-TKO mice (52 ± 2.5 mg/dL) compared to WT mice (72.14 ± 5.4 mg/dL) 24 h after CLP (Figure 3C). There were no significant differences in markers of liver injury, alanine aminotransferase (ALT), and aspartate aminotransferase (AST) in SAA-TKO mice compared to WT mice 24 h after CLP (Figure 3D,E). Consistent with the observation after CLP, the plasma HDL-C levels were not significantly different between WT and SAA-TKO mice following LPS treatment (Figure 3F). There were no apparent differences in the levels of apolipoprotein A1 (apoA1) in the plasma of WT and SAA-TKO mice following sepsis as indicated by western blots (Figure S3A). We analyzed plasma cholesterol 24 h after CLP. There was a modest but significant decrease in total cholesterol in SAA-TKO mice (91.1 ± 12.18 mg/dL) compared to WT mice (128.5 ± 4.938 mg/dL; Figure S3B), and consistent results were obtained when mice were treated with sub-lethal doses of LPS (Figure S3C). 

### 2.4. Deficiency of SAA Exacerbates Sepsis-Induced Kidney and Lung Injury in Mice

**Figure 4:**
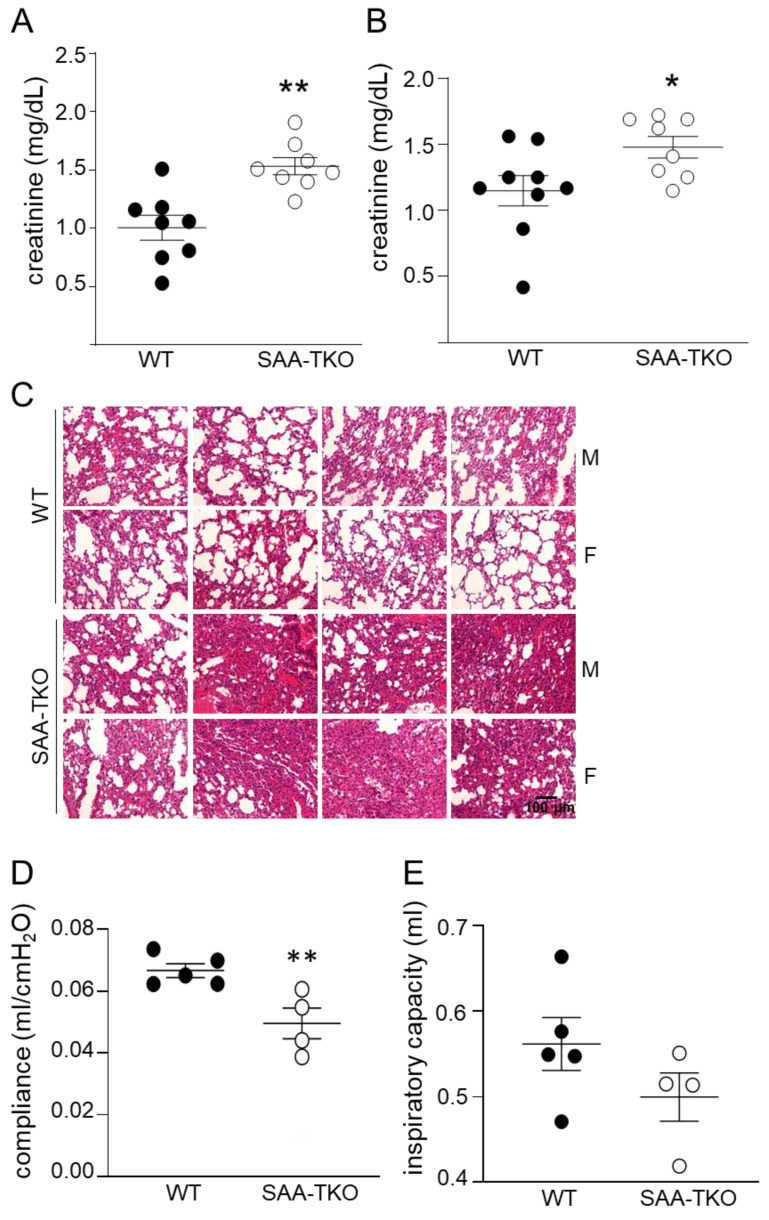
SAA deficiency exacerbates lung injury in sepsis. Plasma creatinine levels in WT and SAA-TKO mice (**A**) 24 h after CLP (**B**) 24 h after a sublethal dose of LPS. (**C**) Representative H&E stained lung sections from male, M, and female, F, WT and SAA-TKO mice (*n* = 4/sex/strain) 24 h after CLP as described under “Section 4”. Scale bar represents 100 μm. (**D**) Compliance and (**E**) inspiratory capacity of the lungs of WT and SAA-TKO mice 24 h after CS treatment, measured using the FlexiVent system as described under “Section 4”. Data are mean ± SEM. * = *p* < 0.05; and ** = *p* < 0.01.

Creatinine, a plasma marker of kidney injury, was significantly higher in SAA-TKO mice (1.53 ± 0.07 mg/dL) than WT mice (1.006 ± 0.11 mg/dL) 24 h following CLP (Figure 4A), implying increased sepsis-induced kidney damage in SAA-TKO mice. Consistent results were obtained when the mice were treated with a sub-lethal dose of LPS (5 mg/kg); plasma creatinine levels 24 h following the LPS injections were significantly higher in SAA-TKO mice compared to WT mice (*p* = 0.03; Figure 4B).

The analysis of lung structure in WT and SAA-TKO mice 24 h after CLP indicated exacerbated lung pathology, including a more pronounced consolidation of lung tissues and atelectasis, in SAA-TKO mice compared to WT mice (Figure 4C). There was no apparent difference in lung tissue architecture between WT and SAA-TKO mice without sepsis (Figure S4). Consistently, SAA-TKO mice showed decreased lung compliance (0.049 ± 0.005 mL/cmH_2_O) compared to WT mice (0.067 ± 0.002 mL/cmH_2_O) (Figure 4D) and a trend for decreased inspiratory capacity (Figure 4E), 24 h after sepsis induction by CS treatment. 

### 2.5. Deficiency of SAA Increases the Expression of Genes Involved in Leukocyte Chemotaxis in Lung Tissues during Sepsis

**Figure 5:**
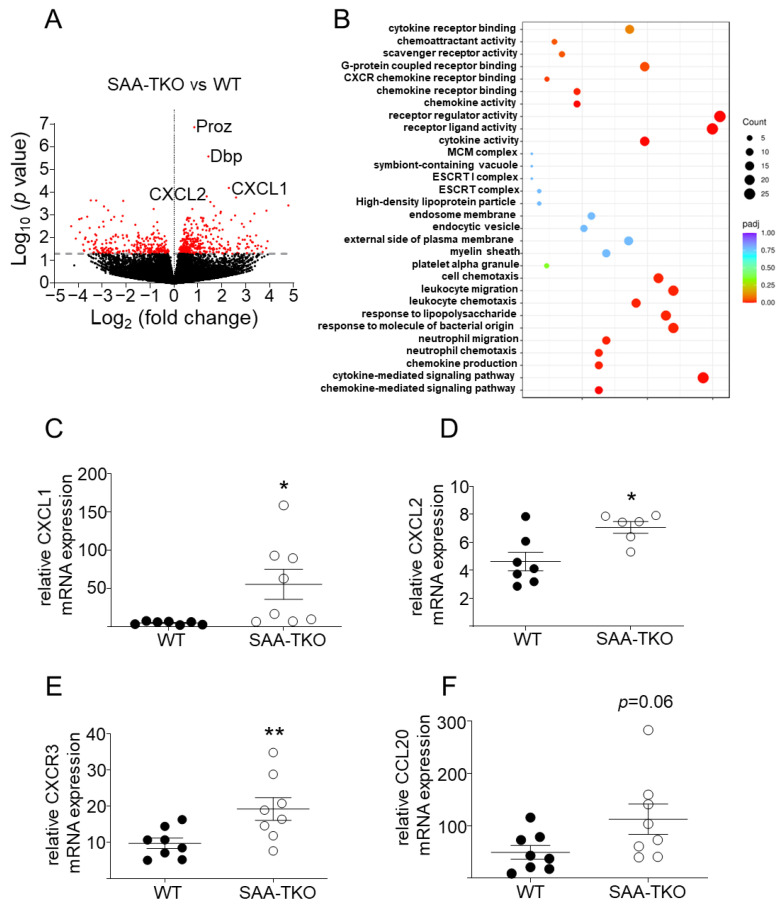
Differentially expressed genes in the lungs of SAA-TKO mice compared to WT mice 24 h following CLP. (**A**) Volcano plot representation of differential gene expression in the lungs of SAA-TKO relative to WT mice 24 h after CLP (*n* = 4/sex/strain). Names of the four highly expressed genes are indicated in the plot. The X-axis shows the fold change in gene expression between different samples, and the Y-axis shows the statistical significance of the differences. (**B**) Gene ontology (GO) pathway analysis of differentially expressed mRNAs. The most significant 30 GO terms are displayed. The size of the point represents the number of genes annotated to a specific GO term, and the changes of color from red to purple represents the significance level of the enrichment. (**C**–**F**) mRNA abundance of CXCL1, CXCL2, CXCR3, and CCL20 in the lung tissues of WT and SAA-TKO mice 24 h after CLP via qPCR as described under “Section 4”. Data are mean ± SEM (*n* = 6–8/strain). * = *p* ≤ 0.05 and ** = *p* ≤ 0.01.

A bulk RNA sequencing analysis of lung tissues excised 24 h after CLP identified several differentially expressed genes (404 upregulated and 260 downregulated) in SAA-TKO mice compared to WT mice (Figure 5A; *p* < 0.05). A gene ontology analysis revealed a significant enrichment of differentially expressed genes associated with chemokine production, chemokine, leukocyte migration, leukocyte chemotaxis, neutrophil chemotaxis, and neutrophil migration, among other pathways, in SAA-TKO lung tissues compared to WT tissues (Figure 5B). Significantly increased expressions of CXCL1 (Figure 5C), CXCL2 (Figure 5D), and CXCR3 (Figure 5E) and a trend of increased expression of CCL20 (Figure 5F) in the lungs of SAA-TKO mice compared to WT mice was confirmed via a qRT-PCR. Thus, SAA deficiency results in exacerbated lung pathology associated with the enhanced expression of genes involved in leukocyte migration and chemotaxis in sepsis in mice. No significant difference in the expression of MCP-1 in the lungs of SAA-TKO mice compared to WT mice was observed (Figure S5).

### 2.6. Deficiency of SAA Increases the Accumulation of Neutrophils in the Lungs of Septic Mice and SAA Attenuates In Vitro Neutrophil Transmigration

**Figure 6:**
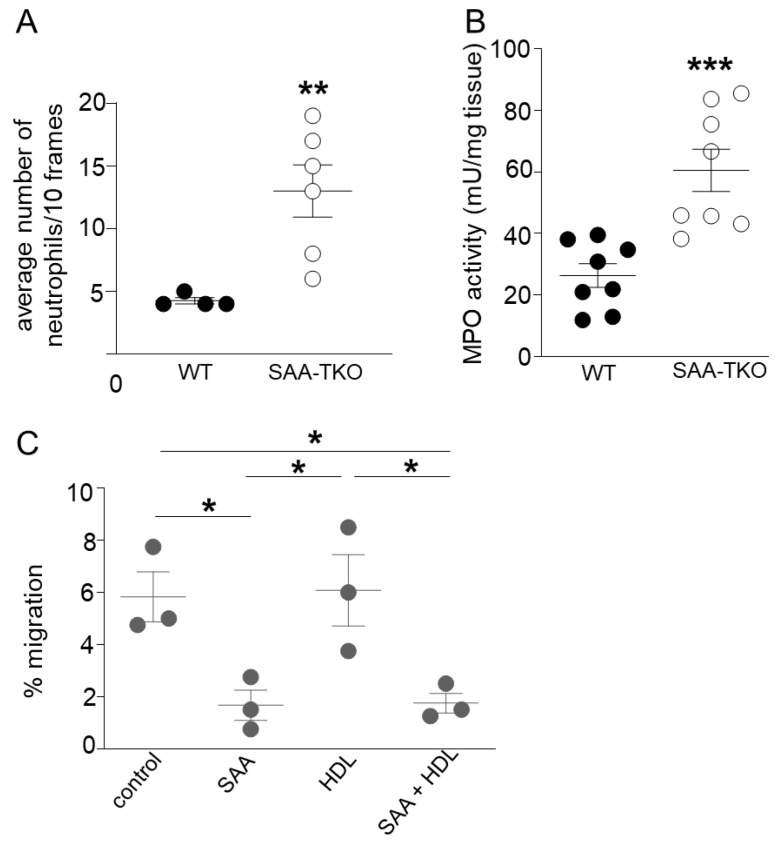
SAA reduces neutrophil accumulation in lungs during sepsis and attenuates neutrophil transmigration in vitro. (**A**) Average neutrophil counts/10 frames (40×) in H&E stained lung tissue sections in WT and SAA-TKO mice, 24 h following CS challenge. Data are mean ± SEM from 4–6 female mice/group. (**B**) Myeloperoxidase activity in lung tissues of WT and SAA-TKO mice 24 h following CLP as described under “Section 4”. Data are mean ± SEM from 4 mice/sex/group. (**C**) A neutrophil transmigration assay was performed as described under “Section 4”. HL-60 cells were incubated with either PBS (control), SAA (5 μg/mL), HDL (14 μg/mL) or SAA + HDL (1:2.8 ratio *w*/*w*) for 1 h at 37 °C before adding to laminin-coated transwells. Cells that migrated to the lower chamber containing 200 nM fMLP were counted and expressed as the percentage of the total cells added. *n* = 3/treatment. * = *p* ≤ 0.05, ** = *p* ≤ 0.01 and *** = *p* ≤ 0.001.

Consistent with the increased expressions of genes involved in neutrophil migration and chemotaxis in the lungs of SAA-TKO mice compared to WT mice in sepsis, there was a significantly increased number of neutrophils (13 ± 2.08 in SAA-TKO mice vs. 4.25 ± 0.25 in WT mice; Figure 6A) associated with significantly increased myeloperoxidase activity (59.51 ± 7.6 mU/mg in SAA-TKO vs. 26.26 ± 3.42 mU/mg in WT; Figure 6B) in the lungs of SAA-TKO mice compared to WT mice in CS-induced sepsis.

As SAA and peptides derived from SAA are known to bind and block the binding of immune cells to the extracellular matrix (ECM) proteins [15,16], we tested whether SAA prevents neutrophil transmigration through laminin in vitro. Differentiated HL-60 (human neutrophil-like cell line) cells were treated with either PBS (control), SAA, an equivalent amount of SAA bound to ahigh-density lipoprotein (SAA-HDL), or HDL as described in Methods. While 5.8 ± 0. 96% of control cells transmigrated into the lower chamber through laminin-coated transwells, a significantly lower percent, only 1.67 ± 0.58% of the SAA treated cells and 1.75 ± 0.38% of the SAA-HDL treated cells, transmigrated into the lower chamber containing chemoattractant N-formyl-methionyl-leucyl-phenylalanine (fMLP; Figure 6C). HDL alone did not significantly impact neutrophil transmigration (6.08 ± 1.37).

## 3. Discussion

Despite the discovery of SAA more than five decades ago and extensive in vitro studies by numerous laboratories, the (patho)physiological functions of SAA remain poorly understood. With the development of SAA knock-out and transgenic mouse models, we are now able to begin to identify the protein’s role in different disease conditions. Studies by our group as well as others have reported that SAA exacerbates the development of chronic inflammatory diseases where the SAA level in circulation is chronically but modestly elevated, such as in diabetes, atherosclerosis, abdominal aortic aneurysm, and cancer [6,17,18,19]. However, SAA levels are increased dramatically but transiently in acute inflammatory diseases where they may play a protective role [10,20]. Our novel data indicate that SAA plays an essential role in resolving acute inflammatory responses and thus enhance survival in sepsis, possibly by ameliorating sepsis-induced lung injury.

SAA is considered a biomarker for sepsis [21,22]. However, only a few studies have investigated whether SAA plays a direct role in sepsis pathogenicity, and no study has evaluated the role of endogenous acute-phase isoforms of SAA. Here, we show that endogenous SAA (all the acute-phase isoforms, collectively referred to as SAA in this manuscript) is protective in sepsis. Earlier studies using a transgenic approach in mice with an inducible expression of human SAA1 in macrophages demonstrated protection against sepsis, LPS-induced inflammation, and acute lung injury [10]. The same group demonstrated that the genetic deletion of *Saa3* rendered mice more susceptible to *P. aeruginosa* infection [20]. SAA3-deficient mice had exacerbated inflammatory responses and more pronounced neutrophil infiltration into the lungs [20]. Using mice deficient in all three acute-phase isoforms of SAA, our study shows that the deficiency of endogenous SAA exacerbates mortality in the three different sepsis models: CLP, treatment with cecal slurry, and endotoxemia (Figure 1). 

Several in vitro studies showed a myriad of proinflammatory activities for SAA [23,24,25,26,27]. SAA in plasma remains predominantly bound to HDL, and emerging literature indicates that many of the proinflammatory effects attributed to SAA are lost when SAA is bound to HDL [6,28,29]. Accumulating evidence indicates that SAA in vivo does not recapitulate the proinflammatory properties reported by in vitro studies, perhaps due to its association with HDL [6,30,31]. Consistently, we did not observe any significant difference in systemic proinflammatory cytokine levels with SAA deficiency in SAA-TKO mice compared to WT mice in sepsis (Figure 2C–F and Figure S2B–D, Supplementary Table S1). While proinflammatory cytokines and chemokines, such as IL-6, TNFα, MCP-1, and IL-8, peak between 2–4 h after the introduction of inflammatory stimuli [32], SAA expression in plasma peaks at ~48 h after the induction of sepsis (Figure S1B) during the resolution phase of acute inflammation [32]. Thus, SAA may play an important role in resolving sepsis. 

In the current study, we did not observe any significant effect of SAA deficiency on plasma endotoxin levels 24 h after CLP or LPS (Figure 2B and Figure S2A). This finding contradicts an earlier report by Cheng et al., [10] where the transgenic expression of human SAA1 in macrophages enhanced endotoxin clearance in septic mice and demonstrated a significant decrease in plasma endotoxin levels in SAA transgenic mice 24 h after CLP or LPS. The difference between the two studies, endogenous SAA vs. forced overexpression in macrophages, could account for the discrepancy in the observations. 

Although an increase in SAA levels in HDL is linked to HDL degradation [33], consistent with our previous reports [13,14], we did not observe any significant changes in plasma HDL-cholesterol (Figure 3A,F) or apoA1 levels (Figure S3A) between WT and SAA-TKO mice in sepsis. SAA is reported to impair the anti-inflammatory properties of HDL [34], thus rendering HDL “dysfunctional”. However, we did not observe any significant differences in systemic inflammatory response between WT and SAA-TKO following sepsis. Among the plasma metabolites analyzed, blood glucose was significantly lower in SAA-TKO compared to WT mice 24 h after CLP but not when treated with LPS, and total cholesterol was modestly but significantly reduced in SAA-TKO mice compared to WT mice in sepsis. Whether the changes observed are the causes or effects of the exacerbated disease is unclear at this point. The liver is the primary site of SAA synthesis during acute inflammation. Interestingly, biomarkers of liver dysfunction, ALT, and AST were not significantly different between SAA-TKO mice and WT mice 24 h after CLP. However, plasma creatinine levels were significantly higher in SAA-TKO mice compared to WT mice 24 h after CLP or LPS treatment, indicating a possibility of exacerbated kidney dysfunction with SAA deficiency. The most striking phenotype of SAA-TKO mice in sepsis is exacerbated lung injury and lung dysfunction. This observation is consistent with reports from other groups on the protective effects of hSAA1 and SAA3 on sepsis-induced lung damage [10,20,35,36]. 

The present study demonstrates the importance of SAA in regulating neutrophil accumulation in the lungs in sepsis. SAA deficiency increases the expression of chemokines and pathways related to leukocyte chemotaxis and migration. Myeloperoxidase activity and the number of neutrophils significantly increased in the lungs of SAA-TKO mice compared to WT mice 24 h after CLP. As maximal SAA expression occurs at ~48 h during the resolution phase of sepsis, this study indicates that SAA may be involved in regulating excessive neutrophil infiltration into the lungs, thus accelerating the resolution process. A coordinated host response is required for successful protection against sepsis. The initiation and resolution of inflammation are active processes that, under optimal conditions, are tightly regulated to achieve the clearance of pathogens and repair of damaged tissues, favoring a return to homeostasis. The complete resolution of an acute inflammatory response is needed for positive outcomes following sepsis. Neutrophils make up a significant part of the defense against pathogens during the early stages of sepsis [37]. For a resolution to ensue, further leukocyte recruitment must be halted and accompanied by the removing of leukocytes from inflammatory sites [38]. Detecting elevated levels of these cells is often associated with the increased clinical deterioration of sepsis [39]. 

In vitro studies indicate that peptides mimicking the sequence of human SAA bind and block the attachment of immune cells, including neutrophils, to ECM proteins [15,16,40]. However, it is unclear whether mouse SAA has such a property. Consistent with all these observations, we show that SAA (both lipid-free and HDL-bound) suppresses the transmigration of HL-60, neutrophil-like cell lines, through laminin-coated-transwells. Thus, SAA may bind to neutrophils and prevent their attachment to the ECM proteins and decrease infiltration into injured tissues. Significantly increased expression of chemokines such as CXCL1, CXCL2, and CXCR3 may also contribute to increased neutrophils in the lungs of SAA-TKO mice compared to WT mice. Further studies are needed to understand SAA’s role in regulating the expression of chemokines in the lungs of septic mice. 

In this investigation, our primary emphasis was on elucidating the pivotal role of SAA in mortality and lung injury subsequent to sepsis. SAA’s potential protective effects may extend beyond the lungs. Previous reports indicate that acute-phase SAAs expressed by the intestinal epithelial cells protect against the development of experimental mouse colitis [41]. Whether SAAs expressed by the gut influence the overall outcomes of the present study needs further investigation. 

In summary, we demonstrate for the first time that the deficiency of endogenous SAA exacerbates lung injury and mortality in sepsis. SAA is expressed during the resolution phase of inflammation, and its deficiency increases neutrophil infiltration into injured lungs. This study also indicates that SAA may bind to neutrophils and prevent their transmigration through ECM proteins into injured lungs. Thus, this study emphasizes the importance of maintaining optimal SAA levels in sepsis treatment. Many treatments for sepsis that target inflammation are expected to reduce plasma SAA levels and thus are counter-productive.

## 4. Materials and Methods

### 4.1. Animals

Mice deficient in SAA1.1, SAA2.1, and SAA3 (SAA1.1/2.1/3-TKO) were generously provided by Drs. June-Yong Lee and Dan Littman, New York University. The SAA1.1/2.1/3-TKO mice were generated by inserting a premature stop codon into exon 2 of *Saa3* in the SAA1.1/2.1-DKO mouse using CRISPR-Cas9 technology as described previously [42]. The wild-type (WT) and SAA-TKO mice (4 months old) used in this study were littermates. The animals were housed in micro-isolator cages and maintained on a 14-h light/10-h dark cycle. The mice were provided with a normal chow diet and water ad libitum. All studies were performed in accordance with the Public Health Service Policy on Humane Care and Use of Laboratory Animals and with the approval of the University of Kentucky Institutional Animal Care and Use Committee.

### 4.2. Mouse Models of Sepsis

Cecal ligation and puncture (CLP)-induced polymicrobial sepsis: CLP was performed as described by Guo et al. [8]. Briefly, mice were anesthetized and an incision of about 1.0 cm was made below the diaphragm. The cecum was isolated, ligated, punctured twice with a needle (22-gauge), and gently pressed to extrude a small amount of cecal material. The cecum was returned to the abdomen, and the muscle and skin incisions were sutured with 6-0 Ethilon suture material to close the incision. The mice were then resuscitated by the injection (s.c.) of 1 mL of phosphate-buffered saline. Sham animals were similarly treated without ligation/puncture of the cecum. 

Cecal slurry (CS)-induced polymicrobial sepsis: CS treatment to induce sepsis was performed as described earlier [9]. CS was prepared by collecting the entire cecal contents from 20-week old C57BL/6 mice as described [43]. The collected cecal contents were combined from the mice, weighed, and mixed with sterile water at (100 mg cecal content: 0.5 mL water). This cecal slurry was sequentially filtered without loss of bacteria and then mixed with an equal volume of 30% glycerol in phosphate-buffered saline (PBS), resulting in a final CS stock solution (1× CS) in 15% glycerol. Each mouse was injected with 300–500 μL of CS intraperitoneally (i.p.) to induce polymicrobial sepsis. The mice received antibiotic treatment (imipenem IPM, 1.5 mg i.p.) and fluid resuscitation (s.c) beginning 12 h after the CS injection and continued twice daily [9]. 

Lipopolysaccharide (LPS)-induced endotoxemia: Mice were injected i.p with either lethal or sublethal doses of LPS derived from *Escherichia coli* 055:B5 (Sigma-Aldrich, St. Louis, MO, USA) diluted in PBS (*w*/*v*), as indicated. The sub-lethal dose for mechanistic studies was 3–5 mg/kg, and the lethal dose for survival studies was 7.5 mg/kg. 

In all three sepsis models, the mice were euthanized at 4 h or 24 h (as indicated in the figure legends) after sepsis induction for mechanistic studies. For survival studies, the mice were monitored for survival until 7 days after sepsis induction.

### 4.3. Cytokine, Glucose, Endotoxin, and Tissue Injury Marker Analysis

Cytokines (IL-6, TNFα, and IL-1β) were quantified with corresponding ELISA kits from R&D systems. Mouse serum cytokines were also analyzed by Eve Technologies (Calgary, AB, Canada) using a mouse cytokine array and chemokine array 31-plex (MD31). Blood glucose was measured according to the manufacturer’s instructions using a blood glucose monitor (Johnson&Johnson, New Brunswick, NJ, USA). The endotoxin levels in plasma were quantitated using an assay kit (GeneScript, Piscataway, NJ, USA). Plasma creatinine was assayed via a colorimetric assay kit (Abcam, Cambridge, UK). The aspartate transaminase (AST) and alanine transaminase (ALT) levels in plasma were determined using assay kits (BioAssay Systems, Hayward, CA, USA).

### 4.4. Analysis of Bacterial Burden

The bacterial burden in blood, peritoneal lavage, and other tissues was determined by a published method [44] with modifications. A 100 µL of blood (1:400 and 1:1600-fold dilutions (*v*/*v*) in PBS), peritoneal lavage (1:400 dilution (*v*/*v*) in PBS), and tissue extracts (60 mg in 1 mL of sterile water, homogenized and diluted (*v*/*v*) 1:400 and 1:1600 times) were plated on BBL^TM^ Brain Heart Infusion (BD, Franklin Lakes, NJ, USA) agar plates. After 24 h incubation at 37 °C, the colonies formed were counted and expressed as CFU/g of tissue or ml of blood or peritoneal lavage. 

### 4.5. Plasma SAA, Total Cholesterol, and Triglyceride Measurements

The plasma SAA concentrations were determined using a mouse SAA ELISA kit (Tridelta Development Ltd., Maynooth, County Kildare, Ireland). The plasma total cholesterol and triglyceride concentrations were measured using enzymatic kits (Wako Chemicals, Richmond, VA, USA).

### 4.6. RNA Isolation and Quantitative RT-PCR

The total RNA was isolated from mouse tissues according to the manufacturer’s instructions (QIAzol reagent, Qiagen, Valencia, CA, USA). The RNA samples were incubated with DNase I (Qiagen, Valencia, CA, USA) for 15 min at RT prior to reverse transcription. The total RNA (0.5–1.0 µg) was reverse transcribed into cDNA using the reverse transcription system (Applied Biosystems, Foster City, CA, USA). After a 4-fold dilution, 5 µL was used as a template for a real-time RT-PCR. Amplification was done for 40 cycles using the Power SYBR Green PCR master Mix Kit (Applied Biosystems, Foster City, CA, USA). The quantification of mRNA was performed using the ΔΔCT method and normalized to GAPDH. The primers used for the quantification of SAA mRNA were designed to recognize all three inducible isoforms of mouse SAA, SAA1.1, SAA2.1, and SAA3 (Forward: GACATGTGGCGAGCCTAC; Reverse: TTGGGGTCTTTGCCACT). The primer sequences for other genes will be provided upon request. 

### 4.7. Histology

The lung tissues from the experimental mice were fixed in 10% formaldehyde, paraffin-embedded, cut into 5-µm sections, and stained with hematoxylin and eosin (Vector Laboratories, Newark, CA, USA). The average neutrophil counts were assessed in H&E stained lung sections in WT and SAA-TKO mice by manually counting the sum of the neutrophils in 10, 40× frames [45] by a pathologist-trained technician in a blinded fashion. An example of a lung section identifying the neutrophils is shown in Figure S6.

### 4.8. Myeloperoxidase (MPO) Activity

The MPO activity in the lungs was determined via a myeloperoxidase activity assay kit (Abcam, Cambridge, UK) following the manufacturer’s instructions. 

### 4.9. Neutrophil Transmigration Assay

A neutrophil transmigration assay was performed as described earlier [46] with modifications. Briefly, transwell plates (24-well, 3-µm pore size; Greiner Bio-One, Monroe, NC, USA) were used for the assay. Insert membranes were pre-coated with 0.5 µg laminin/100 µL/insert for 1–2 h at 37 °C. Human neutrophil-like cell line HL60 cells were differentiated into neutrophils by culturing in RPMI-1640 supplemented with 20%FBS containing DMSO (1.3%) for 5 days. After starving in a 0.2% BSA medium for 16 h, the cells were incubated with human SAA (5 µg/mL), SAA-TKO-HDL (14 µg/mL), or SAA (5 µg/mL) + SAA-TKO-HDL (14 µg/mL) for 1 h at 37 °C. SAA + SAA-TKO-HDL were co-incubated at a ratio of 1:2.8 (protein: protein *w*/*w*) for 1 h at room temperature, a procedure previously shown to efficiently incorporate SAA onto HDL [47], prior to treating the cells. A total of 5 × 10^4^ of the treated cells were transferred to the upper chamber, and the lower chamber was filled with a 0.2% BSA medium containing 200 nM fMLP and incubated at 37 °C for 2 h. Cells that migrated into the lower chamber were collected and counted and expressed as the percentage of total cells added.

### 4.10. Lung Function Measurements

Lung function measurements were undertaken in anesthetized and tracheotomized mice using the FlexiVent system (SCIREQ, Montréal, QC, Canada) as described earlier [48].

### 4.11. RNA Sequencing and Analysis

The total RNA from lung tissues of experimental mice was extracted via the RNeasy Mini Kit according to the manufacturer’s instruction (Qiagen, Valencia, CA, USA) and submitted to the Novogen Corporation Inc. (Sacramento, CA, USA) for RNA-Seq transcriptome sequencing and analysis. RNA purity was checked using a NanoPhotometer, and integrity and quantitation were assessed using the RNA Nano 6000 Assay kit of the Bioanalyzer 2100 system (Agilent Technologies, Santa Clara, CA, USA). A total of 1 µg RNA per sample was used for sample preparation. Sequencing libraries were generated using the NEBNext Ultra ii RNA kit for non-directional libraries. Library quality was assessed using the Agilent Bioanalyzer 21000 system. Sequencing was performed using the Novaseq6000 by Illumina using the S4 flowcell and sequenced at PE150 base pairs. Reads were aligned to the mouse reference genome using Hisat2. The read numbers mapped to each gene were counted using featureCounts v1.5.0-p3. The DESeq2 and edgeR package was used as a differential expression analysis.

### 4.12. Statistics

Data are presented mean ± SEM as indicated in the figure legends. Statistical significance in the experiments comparing two groups was determined by two-tailed Student’s *t*-tests. Normality was tested in all continuous variables by Shapiro-Wilk tests. For the non-repeated continuous data that did not pass the test, the data were analyzed using non-parametric methods. For two-group comparisons, Mann-Whitney U tests were performed. To compare multiple groups, Kruskal-Wallis one-way ANOVAs on Ranks followed by Dunn’s method were used. *p* value < 0.05 was considered to be statistically significant. Survival was analyzed using the logrank test and Kaplan-Meier plots. All experimental data were statistically evaluated with GraphPad Prism 9 software. 

## Data Availability

The data presented in this study are available upon request from the corresponding author.

## Supplementary Materials

The supporting information can be downloaded at: https://www.mdpi.com/article/10.3390/ijms242417501/s1.

## References

[1] Singer M., Deutschman C.S., Seymour C.W., Shankar-Hari M., Annane D., Bauer M., Bellomo R., Bernard G.R., Chiche J.-D., Coopersmith C.M. (2016). The Third International Consensus Definitions for Sepsis and Septic Shock (Sepsis-3). JAMA.

[2] Marshall J.C. (2014). Why have clinical trials in sepsis failed?. Trends Mol. Med..

[3] Vandevyver S., Dejager L., Vandenbroucke R.E., Libert C. (2014). An acute phase protein ready to go therapeutic for sepsis. EMBO Mol. Med..

[4] Kluve-Beckerman B., Drumm M.L., Benson M.D. (1991). Nonexpression of the Human Serum Amyloid A Three (SAA3) Gene. DNA Cell Biol..

[5] Sack G.H. (2018). Serum amyloid A—A review. Mol. Med..

[6] Webb N.R. (2021). High-Density Lipoproteins and Serum Amyloid A (SAA). Curr. Atheroscler. Rep..

[7] Benditt E.P., Eriksen N. (1977). Amyloid protein SAA is associated with high density lipoprotein from human serum. Proc. Natl. Acad. Sci. USA.

[8] Guo L., Song Z., Li M., Wu Q., Wang D., Feng H., Bernard P., Daugherty A., Huang B., Li X.-A. (2009). Scavenger Receptor BI Protects against Septic Death through Its Role in Modulating Inflammatory Response. J. Biol. Chem..

[9] Steele A.M., Starr M.E., Saito H. (2017). Late Therapeutic Intervention with Antibiotics and Fluid Resuscitation Allows for a Prolonged Disease Course with High Survival in a Severe Murine Model of Sepsis. Shock.

[10] Cheng N., Liang Y., Du X., Ye R.D. (2018). Serum amyloid A promotes LPS clearance and suppresses LPS -induced inflammation and tissue injury. EMBO Rep..

[11] Zimetti F., De Vuono S., Gomaraschi M., Adorni M.P., Favari E., Ronda N., Ricci M.A., Veglia F., Calabresi L., Lupattelli G. (2017). Plasma cholesterol homeostasis, HDL remodeling and function during the acute phase reaction. J. Lipid Res..

[12] Tanaka S., Diallo D., Delbosc S., Genève C., Zappella N., Yong-Sang J., Patche J., Harrois A., Hamada S., Denamur E. (2019). High-density lipoprotein (HDL) particle size and concentration changes in septic shock patients. Ann. Intensiv. Care.

[13] de Beer M.C., Webb N.R., Wroblewski J.M., Noffsinger V.P., Rateri D.L., Ji A., van der Westhuyzen D.R., de Beer F.C. (2010). Impact of serum amyloid A on high density lipoprotein composition and levels. J. Lipid Res..

[14] Hosoai H., Webb N.R., Glick J.M., Tietge U.J., Purdom M.S., de Beer F.C., Rader D.J. (1999). Expression of serum amyloid A protein in the absence of the acute phase response does not reduce HDL cholesterol or apoA-I levels in human apoA-I transgenic mice. J. Lipid Res..

[15] Preciado-Patt L., Levartowsky D., Prass M., Hershkoviz R., Lider O., Fridkin M. (1994). Inhibition of cell adhesion to glycoproteins of the extracellular matrix by peptides corresponding to serum amyloid A. Toward understanding the physiological role of an enigmatic protein. JBIC J. Biol. Inorg. Chem..

[16] Urieli-Shoval S., Shubinsky G., Linke R.P., Fridkin M., Tabi I., Matzner Y. (2002). Adhesion of human platelets to serum amyloid A. Blood.

[17] McEneny J., Daniels J.-A., McGowan A., Gunness A., Moore K., Stevenson M., Young I.S., Gibney J. (2015). A Cross-Sectional Study Demonstrating Increased Serum Amyloid A Related Inflammation in High-Density Lipoproteins from Subjects with Type 1 Diabetes Mellitus and How This Association Was Augmented by Poor Glycaemic Control. J. Diabetes Res..

[18] Shridas P., Patrick A.C., Tannock L.R. (2021). Role of Serum Amyloid A in Abdominal Aortic Aneurysm and Related Cardiovascular Diseases. Biomolecules.

[19] Zhou J., Sheng J., Fan Y., Zhu X., Tao Q., He Y., Wang S. (2018). Association between serum amyloid A levels and cancers: A systematic review and meta-analysis. Postgrad. Med. J..

[20] Fan Y., Zhang G., Vong C.T., Ye R.D. (2020). Serum amyloid A3 confers protection against acute lung injury in *Pseudomonas aeruginosa*-infected mice. Am. J. Physiol.-Lung Cell. Mol. Physiol..

[21] Cecconi M., Evans L., Levy M., Rhodes A. (2018). Sepsis and septic shock. Lancet.

[22] Arnon S., Litmanovitz I., Regev R.H., Bauer S., Shainkin-Kestenbaum R., Dolfin T. (2007). Serum amyloid A: An early and accurate marker of neonatal early-onset sepsis. J. Perinatol..

[23] Lee H.Y., Kim S.D., Shim J.W., Lee S.Y., Lee H., Cho K.-H., Yun J., Bae Y.-S. (2008). Serum Amyloid A Induces CCL2 Production via Formyl Peptide Receptor-Like 1-Mediated Signaling in Human Monocytes. J. Immunol..

[24] Patel H., Fellowes R., Coade S., Woo P. (1998). Human Serum Amyloid A has Cytokine-Like Properties. Scand. J. Immunol..

[25] Badolato R., Johnston J.A., Wang J.M., McVicar D., Xu L.L., Oppenheim J.J., Kelvin D.J. (1995). Serum amyloid A induces calcium mobilization and chemotaxis of human monocytes by activating a pertussis toxin-sensitive signaling pathway. J. Immunol..

[26] Baranova I.N., Vishnyakova T.G., Bocharov A.V., Kurlander R., Chen Z., Kimelman M.L., Remaley A.T., Csako G., Thomas F., Eggerman T.L. (2005). Serum Amyloid A Binding to CLA-1 (CD36 and LIMPII Analogous-1) Mediates Serum Amyloid A Protein-induced Activation of ERK1/2 and p38 Mitogen-activated Protein Kinases. J. Biol. Chem..

[27] Song C., Hsu K., Yamen E., Yan W., Fock J., Witting P.K., Geczy C.L., Ben Freedman S. (2009). Serum amyloid A induction of cytokines in monocytes/macrophages and lymphocytes. Atherosclerosis.

[28] Badolato R., Wang J.M., Murphy W.J., Lloyd A.R., Michiel D.F., Bausserman L.L., Kelvin D.J., Oppenheim J.J., Fava R.A., Olsen N.J. (1994). Serum amyloid A is a chemoattractant: Induction of migration, adhesion, and tissue infiltration of monocytes and polymorphonuclear leukocytes. J. Exp. Med..

[29] Franco A.G., Sandri S., Campa A. (2011). High-density lipoprotein prevents SAA-induced production of TNF-α in THP-1 monocytic cells and peripheral blood mononuclear cells. Memórias Inst. Oswaldo Cruz.

[30] Witting P.K., Song C., Hsu K., Hua S., Parry S.N., Aran R., Geczy C., Freedman S.B. (2011). The acute-phase protein serum amyloid A induces endothelial dysfunction that is inhibited by high-density lipoprotein. Free Radic. Biol. Med..

[31] Simons J.P., Al-Shawi R., Ellmerich S., Speck I., Aslam S., Hutchinson W.L., Mangione P.P., Disterer P., Gilbertson J.A., Hunt T. (2013). Pathogenetic mechanisms of amyloid A amyloidosis. Proc. Natl. Acad. Sci. USA.

[32] Bannenberg G.L., Chiang N., Ariel A., Arita M., Tjonahen E., Gotlinger K.H., Hong S., Serhan C.N. (2005). Molecular Circuits of Resolution: Formation and Actions of Resolvins and Protectins. J. Immunol..

[33] Cho K.-H. (2022). Human Serum Amyloid a Impaired Structural Stability of High-Density Lipoproteins (HDL) and Apolipoprotein (Apo) A-I and Exacerbated Glycation Susceptibility of ApoA-I and HDL. Molecules.

[34] Han C.Y., Tang C., Guevara M.E., Wei H., Wietecha T., Shao B., Subramanian S., Omer M., Wang S., O’Brien K.D. (2016). Serum amyloid A impairs the antiinflammatory properties of HDL. J. Clin. Investig..

[35] Ather J.L., Dienz O., Boyson J.E., Anathy V., Amiel E., Poynter M.E. (2018). Serum Amyloid A3 is required for normal lung development and survival following influenza infection. Sci. Rep..

[36] Renckens R., Roelofs J.J.T.H., Knapp S., de Vos A.F., Florquin S., van der Poll T. (2006). The Acute-Phase Response and Serum Amyloid A Inhibit the Inflammatory Response to *Acinetobacter baumannii* Pneumonia. J. Infect. Dis..

[37] Shen X.F., Cao K., Jiang J.P., Guan W.X., Du J.F. (2017). Neutrophil dysregulation during sepsis: An overview and update. J. Cell. Mol. Med..

[38] Spite M., Serhan C.N. (2010). Novel Lipid Mediators Promote Resolution of Acute Inflammation. Circ. Res..

[39] Jarczak D., Kluge S., Nierhaus A. (2021). Sepsis—Pathophysiology and Therapeutic Concepts. Front. Med..

[40] Preciado-Patt L., Pras M., Fridkin M. (1996). Binding of human serum amyloid A (hSAA) and its high-density Iipoprotein3 complex (hSAA-HDL3) to human neutrophils. Possible implication to the function of a protein of an unknown physiological role. Int. J. Pept. Protein Res..

[41] Zhang G., Liu J., Wu L., Fan Y., Sun L., Qian F., Chen D., Ye R.D. (2018). Elevated Expression of Serum Amyloid A 3 Protects Colon Epithelium Against Acute Injury Through TLR2-Dependent Induction of Neutrophil IL-22 Expression in a Mouse Model of Colitis. Front. Immunol..

[42] Lee J.-Y., Hall J.A., Kroehling L., Wu L., Najar T., Nguyen H.H., Lin W.-Y., Yeung S.T., Silva H.M., Li D. (2019). Serum Amyloid A Proteins Induce Pathogenic Th17 Cells and Promote Inflammatory Disease. Cell.

[43] Starr M.E., Steele A.M., Saito M., Hacker B.J., Evers B.M., Saito H. (2014). A New Cecal Slurry Preparation Protocol with Improved Long-Term Reproducibility for Animal Models of Sepsis. PLoS ONE.

[44] Speer E.M., Diago-Navarro E., Ozog L.S., Raheel M., Levy O., Fries B.C. (2020). A Neonatal Murine Escherichia coli Sepsis Model Demonstrates That Adjunctive Pentoxifylline Enhances the Ratio of Anti- vs. Pro-inflammatory Cytokines in Blood and Organ Tissues. Front. Immunol..

[45] Matute-Bello G., Downey G., Moore B.B., Groshong S.D., Matthay M.A., Slutsky A.S., Kuebler W.M. (2011). An Official American Thoracic Society Workshop Report: Features and Measurements of Experimental Acute Lung Injury in Animals. Am. J. Respir. Cell Mol. Biol..

[46] Li S.-C., Tsai K.-W., Huang L.-H., Weng K.-P., Chien K.-J., Lin Y., Tu C.-Y., Lin P.-H. (2020). Serum proteins may facilitate the identification of Kawasaki disease and promote in vitro neutrophil infiltration. Sci. Rep..

[47] Shridas P., De Beer M.C., Webb N.R. (2018). High-density lipoprotein inhibits serum amyloid A–mediated reactive oxygen species generation and NLRP3 inflammasome activation. J. Biol. Chem..

[48] Valenca S.S., Dong B.E., Gordon E.M., Sun R.C., Waters C.M. (2022). ASK1 Regulates Bleomycin-induced Pulmonary Fibrosis. Am. J. Respir. Cell Mol. Biol..

