# Supplementary material for: Deficiency of Acute-Phase Serum Amyloid A Exacerbates Sepsis-Induced Mortality and Lung Injury in Mice
Source: Int J Mol Sci. 2023 Dec 15;24(24):17501. doi: 10.3390/ijms242417501 (PMC10744229; doi:10.3390/ijms242417501)
Supplement: Supplementary file 1 [file ijms-24-17501-s001.zip › Ji et al. spplemental figure 1.pdf]

Figure S1

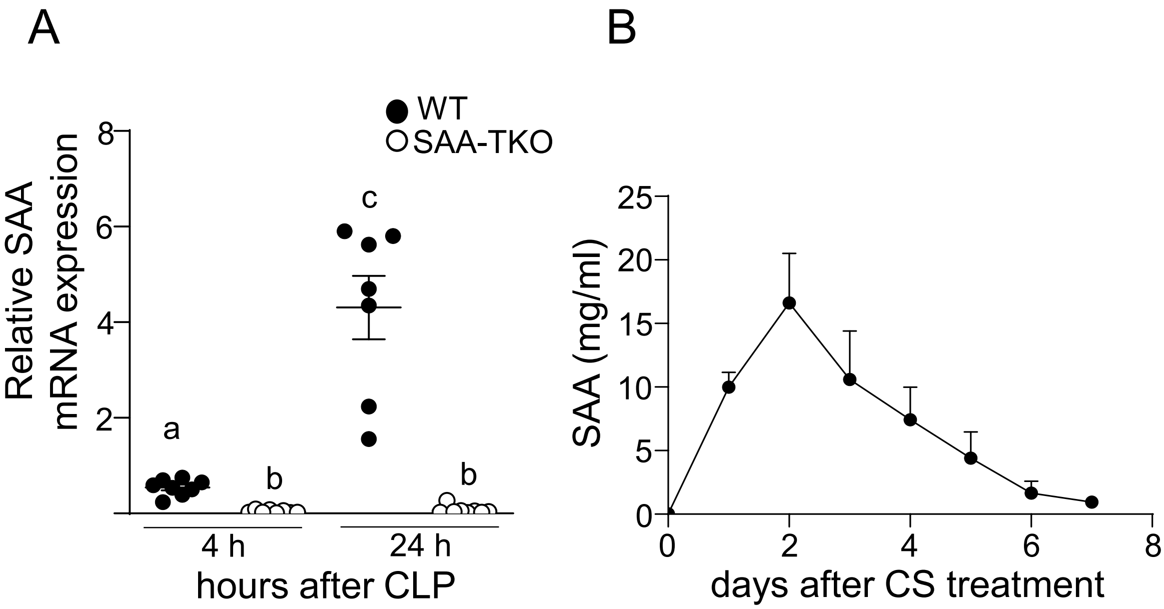

Supplemental Figure 1. SAA expression in sepsis. (A) SAA mRNA abundance in the livers of WT and SAA-TKO mice (male and female) after 4 h and 24 h following CLP-induced sepsis by qPCR. The primers were designed to detect all three inducible SAA isoforms, SAA 1.1, SAA 2.1 and SAA3 as described under "Materials and Methods". In A, groups that are not significantly different ($p \geq 0.05$) are indicated with the same letter. (B) Plasma SAA concentrations determined by Elisa as described under "Materials and Methods" in male C57Bl/6 mice (n=7; mean ±SEM) up to 7 days after cecal slurry treatment.
