# Supplementary material for: Deficiency of Acute-Phase Serum Amyloid A Exacerbates Sepsis-Induced Mortality and Lung Injury in Mice
Source: Int J Mol Sci. 2023 Dec 15;24(24):17501. doi: 10.3390/ijms242417501 (PMC10744229; doi:10.3390/ijms242417501)
Supplement: Supplementary file 1 [file ijms-24-17501-s001.zip › Ji et al. spplemental figure 2.pdf]

Figure S2

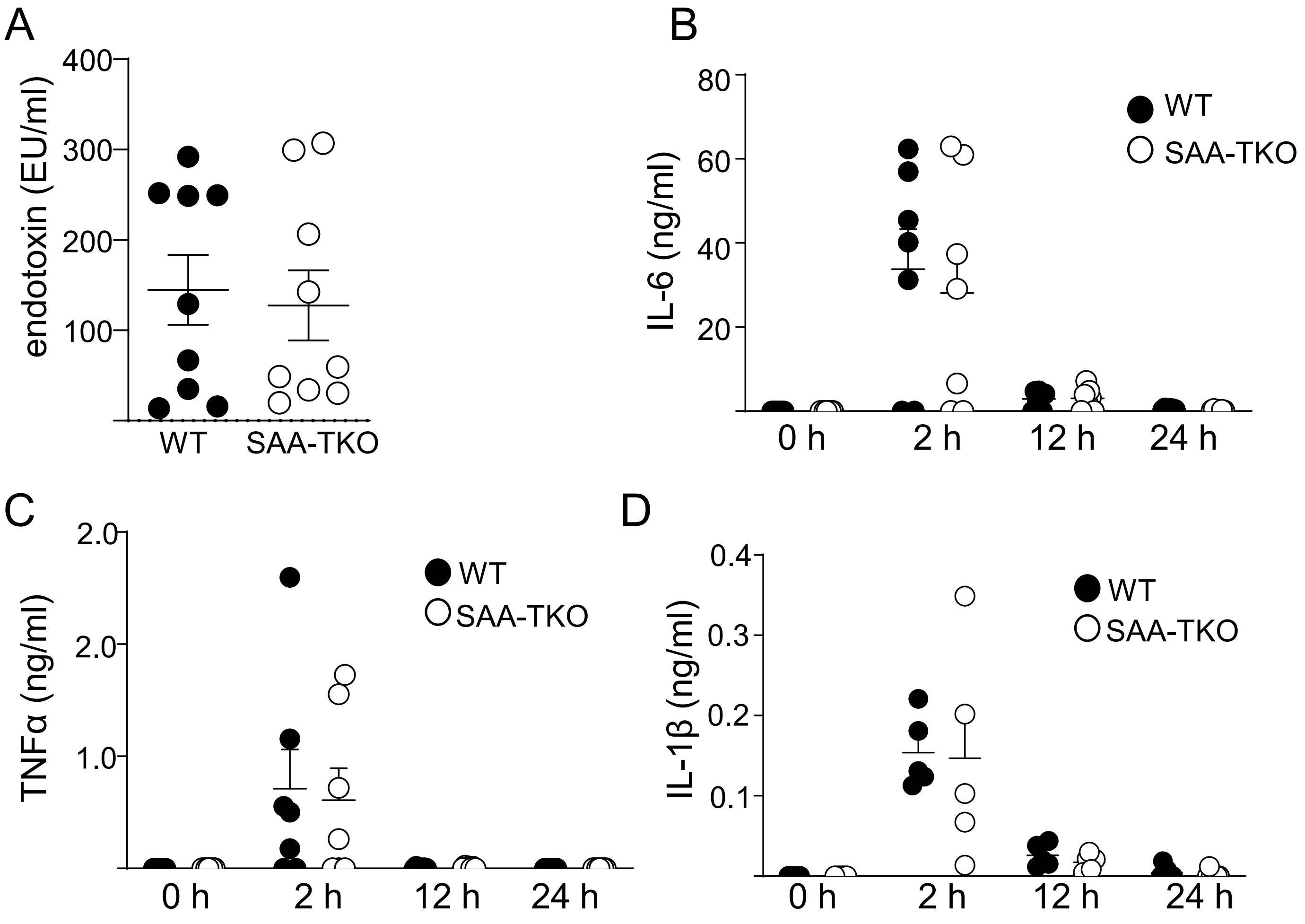

Supplemental Figure 2. SAA deficiency does not significantly alter endotoxin clearance or plasma cytokine levels following LPS treatment. (A) plasma endotoxin levels in WT and SAA-TKO mice (n=9/strain) 24 h after treatment with LPS, determined as described under "Materials and Methods". (B-D) plasma IL-6, TNFα and IL-1β by ELISA in WT and SAA-TKO mice (n=8/strain) at 0 h, 2 h, 12 h and 24 h after LPS treatment, determined as described under "Materials and Methods".
