# Supplementary material for: Deficiency of Acute-Phase Serum Amyloid A Exacerbates Sepsis-Induced Mortality and Lung Injury in Mice
Source: Int J Mol Sci. 2023 Dec 15;24(24):17501. doi: 10.3390/ijms242417501 (PMC10744229; doi:10.3390/ijms242417501)
Supplement: Supplementary file 1 [file ijms-24-17501-s001.zip › Ji et al. spplemental figure 3.pdf]

Figure S3

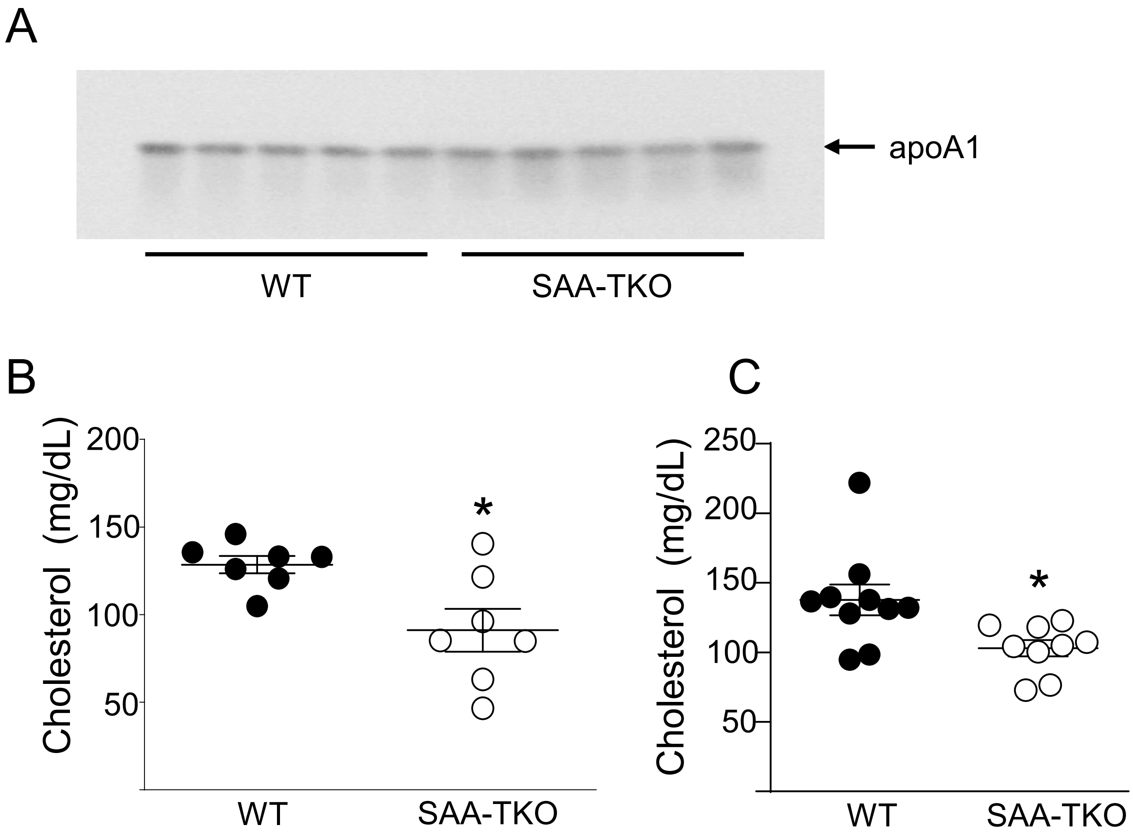

Supplemental Figure 3. SAA deficiency does not alter plasma apoA1 levels following sepsis. (A) Plasma apoA1 levels were analyzed by western blot in WT and SAA-TKO mice (n=5/strain) 24 h after CLP treatment. Plasma total cholesterol levels after (B) CLP and (C) LPS treatment. Data are mean ±SEM, *= $p<0.05$.
