# Supplementary material for: Deficiency of Acute-Phase Serum Amyloid A Exacerbates Sepsis-Induced Mortality and Lung Injury in Mice
Source: Int J Mol Sci. 2023 Dec 15;24(24):17501. doi: 10.3390/ijms242417501 (PMC10744229; doi:10.3390/ijms242417501)
Supplement: Supplementary file 1 [file ijms-24-17501-s001.zip › Ji et al. spplemental figure 4.pdf]

Figure S4

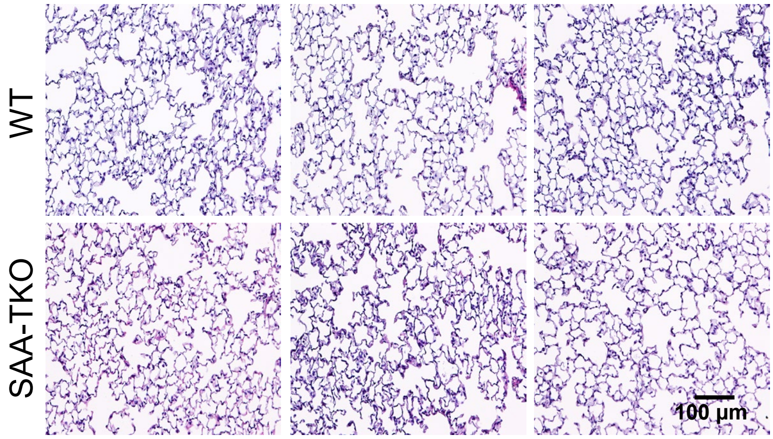

Supplemental Figure 4. Representative H&E stained lung sections from normal control male WT and SAA-TKO mice. The lung tissues were excised from control untreated WT and SAA-TKO mice (n=3/strain) and stained with H&E as described under “Materials and Methods.” The scale bar represents 100 µm.
