# Supplementary material for: Deficiency of Acute-Phase Serum Amyloid A Exacerbates Sepsis-Induced Mortality and Lung Injury in Mice
Source: Int J Mol Sci. 2023 Dec 15;24(24):17501. doi: 10.3390/ijms242417501 (PMC10744229; doi:10.3390/ijms242417501)
Supplement: Supplementary file 1 [file ijms-24-17501-s001.zip › Ji et al. spplemental figure 5.pdf]

Figure S5

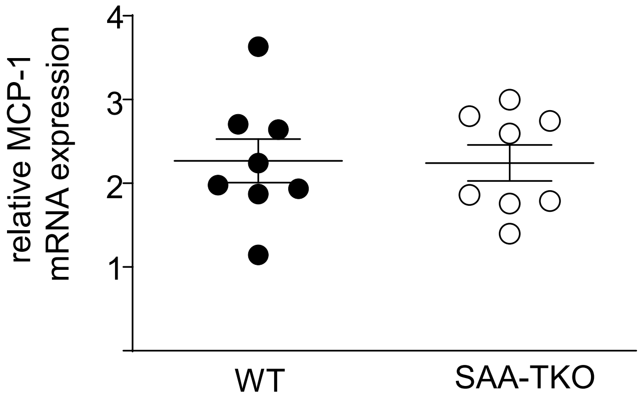

Supplemental figure 5. Deficiency of SAA does not significantly impact lung MCP-1 expression. Lung MCP-1 mRNA abundance in the lung tissues of WT and SAA-TKO mice 24 h after CLP by qPCR. Data are mean ±SEM.
