# Supplementary material for: Deficiency of Acute-Phase Serum Amyloid A Exacerbates Sepsis-Induced Mortality and Lung Injury in Mice
Source: Int J Mol Sci. 2023 Dec 15;24(24):17501. doi: 10.3390/ijms242417501 (PMC10744229; doi:10.3390/ijms242417501)
Supplement: Supplementary file 1 [file ijms-24-17501-s001.zip › Ji et al. spplemental figure 6.pdf]

Figure S6

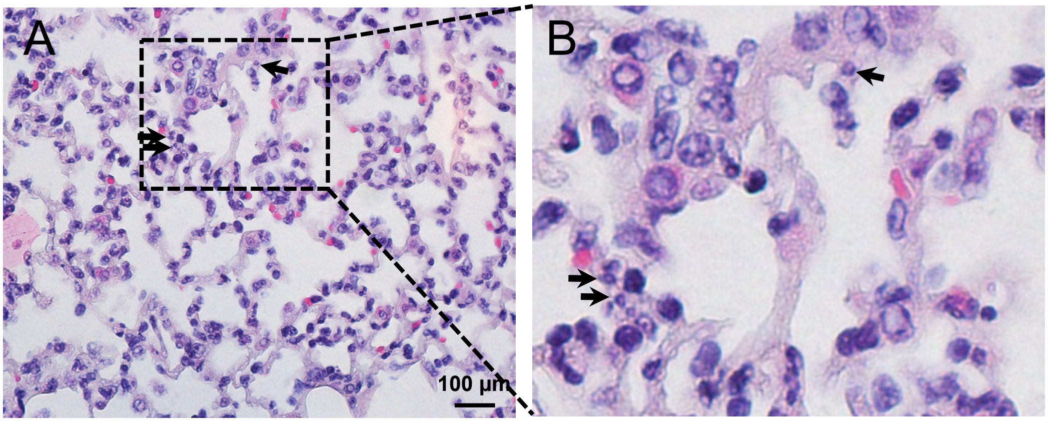

Supplemental figure 6. Identification of neutrophils in lung tissues following sepsis-induced lung injury. (A) Neutrophils (black arrow) in H&E stained lung tissue sections from a mouse with lung injury 24 h following CS challenge. Images taken at 40X magnification. (B) Further magnification from the section in A. Scale bar in (A) represents 100 µm.
