# Supplementary material for: Deficiency of Acute-Phase Serum Amyloid A Exacerbates Sepsis-Induced Mortality and Lung Injury in Mice
Source: Int J Mol Sci. 2023 Dec 15;24(24):17501. doi: 10.3390/ijms242417501 (PMC10744229; doi:10.3390/ijms242417501)
Supplement: Supplementary file 1 [file ijms-24-17501-s001.zip › Supplementary table 1.pdf]

Supplementary table 1: Serum concentrations of cytokines in WT and SAA-TKO mice following CLP

	4h post CLP; pg/ml (mean±SEM)		p-value	24h post CLP; pg/ml (mean±SEM)		p-value
	WT	SAA-TKO				
VEGF	2.099±0.22	2.315±0.39	>0.9999	2.183±0.93	2.27±0.80	>0.9999
RANTES	89.23±8.2	118.6±22.8	>0.9999	191.1±147	97.23±43.3	>0.9999
MIP-2	2536±480.4	4917±1835	0.3003	192.9±16.9	2411±1827	0.5451
MIP-1β	2129±532	4758±1522	0.136	1558±1447	1475±825	>0.9999
MIP-1α	690.1±103	1389±381	>0.9999	2372±2162	1059±649	0.9996
MIG	197±45.2	266.7±88.7	>0.9999	203.5±85.9	186.7±32.7	>0.9999
MCSF	17.67±3.2	26.18±4.9	>0.9999	67.28±55.5	60.51±25.4	>0.9999
LIX	4994±496	4314±375	>0.9999	3140±575	2220±434	>0.9999
LIF	22.74±3.6	49.58±12.7	>0.9999	110±106.6	51.63±29.3	>0.9999
IP-10	424.5±39	604.6±96.2	>0.9999	243.8±72.6	160.5±30.2	>0.9999
IL-17	127.4±24.7	378.9±120.8	>0.9999	7.624±1.3	22.9±5.6	>0.9999
IL-15	78.56±13.8	175.8±102.7	>0.9999	111.6±16.2	101.3±35.7	>0.9999
IL-13	236.6±18.8	261.8±14.7	>0.9999	109.2±37.8	124±34.4	>0.9999
IL-12(p70)	83.59±14.3	81.86±17.7	>0.9999	50.33±13.7	47.2±15.9	>0.9999
IL-12(p40)	8.456±1.8	12.02±1.9	>0.9999	9.287±3.1	14.54±4.4	>0.9999
IL-10	2020±542.8	4870±2094	0.0605	2829±2593	5249±2511	0.2677
IL-9	194±21.6	170.7±21.1	>0.9999	109.9±18.6	173.4±60.0	>0.9999
IL-7	10.04±3.5	12.13±6.2	>0.9999	12.53±3.0	7.87±2.8	>0.9999
IL-5	265.4±35.9	317.2±68.2	>0.9999	31.09±25.0	14.38±3.3	>0.9999
IL-4	2.836±1.2	1.713±0.4	>0.9999	0.6271±0.1	0.804±0.3	>0.9999
IL-3	2.061±0.2	2.613±0.3	>0.9999	2.494±0.7	3.135±0.7	>0.9999
IL-2	19.1±2.7	17.09±2.5	>0.9999	20.36±4.9	22.93±6.9	>0.9999
IL-1α	815.9±54.8	835.6±94.3	>0.9999	827.2±157.3	761±64.1	>0.9999
IFNγ	5.538±1.3	4.545±0.7	>0.9999	3.75±1.5	3.241±1.1	>0.9999
GM-CSF	20.38±1.3	29.33±5.3	>0.9999	50.73±35.7	19.74±3.3	>0.9999
G-CSF	31142±901.5	29453±1603	0.9501	32207±101.4	32717±150.4	>0.9999
Eotaxin	1725±137.4	1999±259.1	>0.9999	1458±428.4	754.3±89.8	>0.9999
